# The Viborg vascular (VIVA) screening trial of 65-74 year old men in the central region of Denmark: study protocol

**DOI:** 10.1186/1745-6215-11-67

**Published:** 2010-05-27

**Authors:** Nikolaj Grøndal, Rikke Søgaard, Eskild W Henneberg, Jes S Lindholt

**Affiliations:** 1 Department of vascular surgery, Viborg Hospital, Denmark; 2 Centre for Health Services Research and Technology Assessment (CAST), University of Southern Denmark

## Abstract

**Background:**

Screening for abdominal aortic aneurysm (AAA) of men aged 65-74 years reduces the AAA-related mortality and is generally considered cost effective. Despite of this only a few national health care services have implemented permanent programs.

Around 10% of men in this group have peripheral arterial disease (PAD) defined by an ankle brachial systolic blood pressure index (ABI) below 0.9 resulting in an increased mortality-rate of 25-30%. In addition well-documented health benefits may be achieved through primary prophylaxis by initiating systematic cholesterol-lowering, smoking cessation, low-dose acetylsalicylic acid (aspirins), exercise, a healthy diet and blood-pressure control altogether reducing the increased risks for cardiovascular disease by at least 20-25%.

The benefits of combining screening for AAA and PAD seem evident; yet they remain to be established. The objective of this study is to assess the efficacy and the cost-effectiveness of a combined screening program for AAA, PAD and hypertension.

**Methods:**

The Viborg Vascular (VIVA) screening trial is a randomized, clinically controlled study designed to evaluate the benefits of vascular screening and modern vascular prophylaxis in a population of 50,000 men aged 65-74 years. Enrolment started October 2008 and is expected to stop in October 2010. The primary outcome is all-cause mortality. The secondary outcomes are cardiovascular mortality, AAA-related mortality, hospital services related to cardiovascular conditions, prevalence of AAA, PAD and potentially undiagnosed hypertension, health-related quality of life and cost effectiveness. Data analysis by intention to treat.

**Results:**

Major follow-up will be performed at 3, 5 and 10 years and final study result after 15 years.

**Trial registration:**

ClinicalTrials.gov NCT00662480

## Background

It has been demonstrated that abdominal aortic aneurysm (AAA) screening of men aged 65-74 years reduces AAA mortality. Research at Viborg Hospital published in the British Medical Journal in March 2005 [1] shows that only 352 Danish men above the age of 65 require AAA screening to avoid one AAA rupture-related death in the subsequent 5-year period. Furthermore, it seems that the long-term economic efficiency of screening is at a level, which is generally considered cost effective, at least from a health care sector perspective [2-4]. Despite the clinical and economic evidence for the attractiveness of population screening, few national health care services have implemented permanent programs.

The majority of AAAs diagnosed by screening does not comprise a rupture risk due to being detected early, but nearly half expand to sizes requiring repair. However growth may be limited through smoking cessation [5,6], low-dose aspirin [7-9], statin treatment [10] and ACE inhibitor therapy [11] which will ultimately also reduce the increased cardiovascular risk of the affected patients [12].

International studies indicate that approximately 5-10% of men above the age of 60 show signs of peripheral arterial disease (PAD; ankle brachial systolic blood pressure index (ABI) <0.9) - a proportion which increases with age, and mainly non-symptomatic. Approximately 25-30% of these men will die from cardiovascular disease within a 5-year period, and an even higher proportion will need hospitalization due to cardiovascular disease. Cholesterol-lowering, smoking cessation, low-dose acetylsalicylic acid (aspirins), exercise, a healthy diet and blood-pressure control reduce the increased risks for cardiovascular disease by at least 20-25% [13-16]. So far, screening studies have neglected the interaction between PAD-screening and such general prophylactic efforts, and thus the benefit and cost effectiveness of screening may be underestimated.

In sum, well-documented health benefits may be achieved through prophylactic screening for cardiovascular disease. Advantages include fewer premature deaths and a reduction in the number of amputations and hospital admissions. Furthermore, hospital costs may be reduced by the decrease in admissions and in-hospital post-operative care.

The benefits of combining screening for AAA and PAD thus seem evident; yet they remain to be established. The objective of this study is to assess the efficacy and the cost-effectiveness of a combined screening program for AAA, PAD and hypertension.

## Methods

### The Viborg Vascular screening program

**Figure 1:**
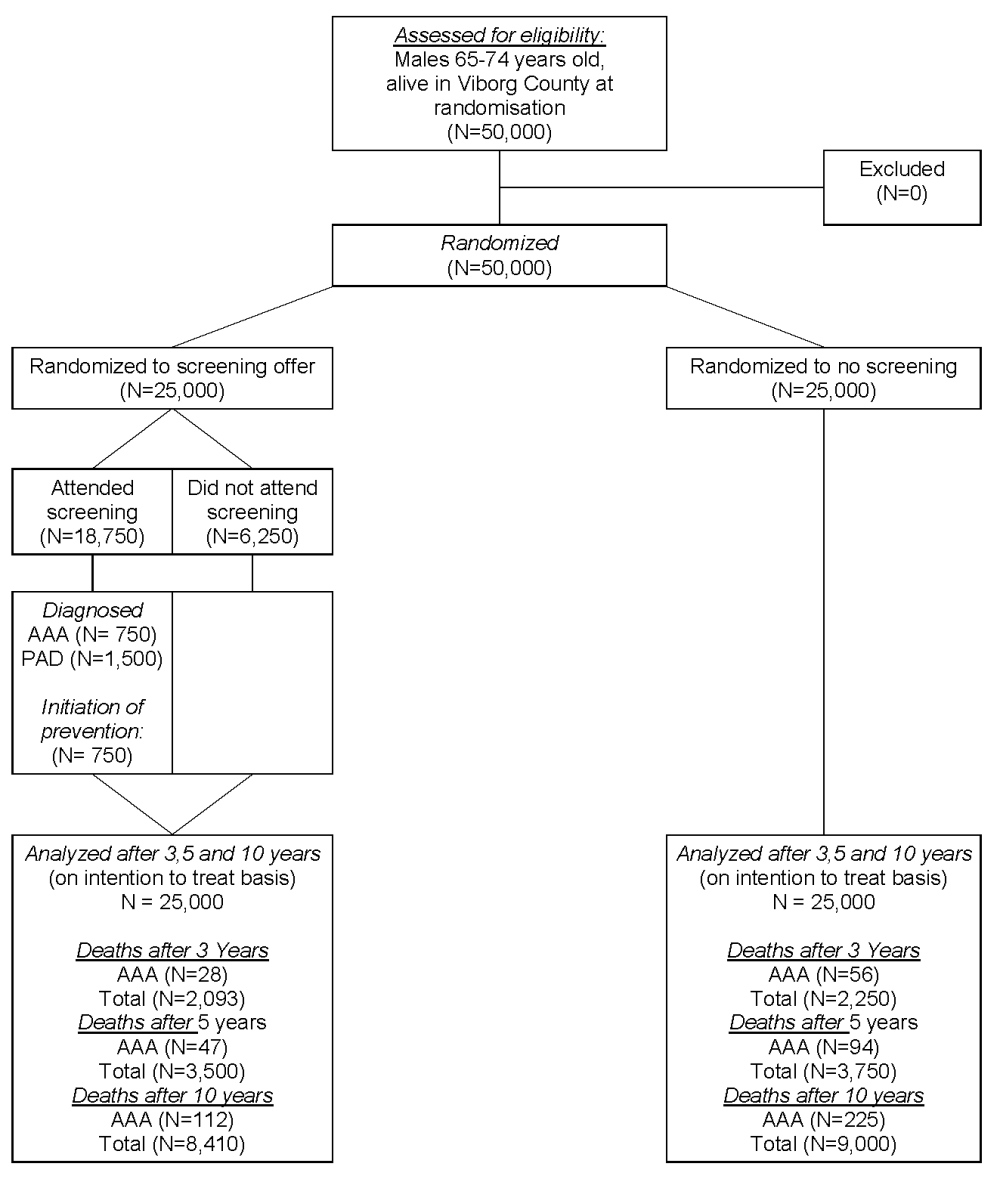
**Expected flow-chart concerning vascular screening (VIVA) for peripheral arterial disease, abdominal aortic aneurysm and hypertension**.

In the timeframe of October 2008 until October 2010 approximately 50,000 65-74 year-old Danish men will be randomized to either receive an invitation for vascular screening or being a control (Figure 1). ABI measurement, ultrasound scan of the aorta in addition to a questionnaire regarding lifestyle parameters, medical and smoking status is performed by trained project-nurses in 3 mobile units at local hospitals in the mid region of Jutland, Denmark.

In the case of infrarenal aortic dilatation such findings are; i) pre-aneurysmatic (<24-30 mm<) or ii) aneurysmatic (≥30 mm). Dilatation <50 mm is controlled annually with ultrasound scanning and dilatation > 50 mm referred for consultation by a vascular surgeon. PAD may be reliable diagnosed with a portable ultrasound Doppler and blood pressure cuff. PAD is defined as ABI below 0.9 or above 1.4 and annual control is offered. In the case of suspected undiscovered hypertension (bloodpressure > 160/100) the patient is encouraged to see his family physician for further assessment and treatment.

During a training course, six project-nurses have been educated in the natural history of AAA, PAD and hypertension, instruction in life style changes and smoking cessation, blood sampling and bed side cholesterol measurement and the initiation of antiplatelet and lipid lowering medication. They were trained under supervision to measure ABI and perform ultrasound scanning of the aorta Independent examinations were only allowed, when the team-interobserver variation of aortic anterior-posterior diameter were below 2 mm, and ABI below 10%. The nurses operate in three mobile teams which will each be equipped with uniform portable Dopplers, blood pressure cuffs and portable ultrasound scanners and perform the screening sessions at the hospitals in the region. The project secretary will continually invite participants to their regional hospital using the names and address information from the randomization files.

Participants are informed that they should wear expedient footwear. The letter is accompanied by an option to refuse or reschedule in writing. Non-responders are re-invited once. Finally, enclosed with the invitation is a small questionnaire on smoking habits, anamnesis, walking-related pain (Walking Impairment Questionnaire), use of medicine, and the EUROQOL five dimensions (EQ5D) health questionnaire to obtain psychometric measures. This has recently been validated in Danish population norms [17]. Study participants commit to the program with a written consent using pre-printed consent forms.

To visualise the aorta the ultrasound 4 MHz transducer is placed longitudinally just above and a little to the left of the navel. In cases of dilation, the maximal perpendicular AP diameter is measured. If no dilation is found, the AP diameter is measured two centimetres above the bifurcation. Simultaneously, peripheral pulse data are recorded by the other nurse. Brachial blood pressure is automatically measured concurrently with the ankle blood pressure by detecting the Doppler signal from the dorsalis pedis artery (DPA) and first inflating the blood pressure cuff until the signal disappears and then deflating the cuff until the signal reappears. The mean pressure of the two measurements is recorded, and the same procedure performed for the tibialis posterior artery (TPA). Subsequently, the same procedure is repeated in the opposite extremity.

### Follow-up and data collection

All positive findings are subject to clinical monitoring to provide a reassuring context for the communication of the finding and its consequences. In the case of PAD the participant is informed of the relatively positive prognosis of leg survival and the need to initiate suitable prophylactic measures including walking/exercise, smoking cessation, low-fat diet, aspirin and statin treatment. Cholesterol levels are measured. If it exceeds 4.0 mmol/l, cholesterol is unlikely to fall below 3.5 mmol/l following relevant diet changes and, consequently, the patient is given a prescription for 40 mg simvastatin/day. Annual follow-up with ABI measurement is offered. The EQ-5D is fulfilled upon visit and in connection with any follow-up.

In the case of AAA the aorta is reassessed, the finding is stored digitally and the calcification degree and mural thrombus is determined. Information regarding the natural history of AAA is delivered and the need to initiate suitable prophylactic measures including exercise, smoking cessation, low-fat diet, aspirin and statin treatment in accordance with the guideline from the European Society of Cardiology 2007. If the AAA exceeds a diametre of ≥ 50 mm, the patient is referred for CT scan and assessment by a vascular surgeon to further surveillance or surgery. If the AAA is below 5 cm, annual control with ultrasound scan is offered. The EQ-5D is fulfilled upon visit and in connection with any follow-up, as well as annually after AAA surgery.

Data from the screening procedure are collected in a large database. Information concerning deaths, including-date and cause of death, is obtained from the Civil Registration System. Information on visits to outpatient clinics and hospital admissions caused by cardiovascular conditions including amputations is obtained from the National Patient Registry. From the Danish Causes of Death Registry, data on cause of death are collected. The information is classified according to cause; AAA-related or cardiovascular.

### Study population

To maximize the possibility of detecting an effect, men aged 65-74 are screened, as this group has a high prevalence of non-symptomatic cardiovascular disease as well as a long remaining life expectancy. Screening of men above the age of 74 presumably would have resulted in higher prevalences of AAA and PAD, but also a higher mortality associated with other conditions and a lower acceptance rate. Similarly, screening of men below the age of 65 would demonstrate lower mortality, but also a lower prevalence and a lower rupture frequency. Among women in the age interval of 65-74, PAD and AAA frequencies are reported to be lower and to occur later in life; hence screening women would, ceteris paribus, yield a lower detection rate. Furthermore, as mortality from other causes is even higher among women as compared to men, the effect of detecting a case would be inferior. It should be noted however that the reports of such differences between genders are old and the extent to which significant differences persist is uncertain.

As per January 1, 2007 there were approximately 50,000 men aged 65-74 years in the Mid Region of Denmark, which is located from coast to coast in the middle of Jutland. This population comprise the target group for the study which has no exclusion criteria.

### Randomization

Civil registration numbers and patient data are obtained from national registries. Randomisation is stratified by municipalities, as the prevalences of the diseases are expected to differ in this relatively large region. The Region consists of 19 counties covering more than 13,000 km2, which includes some of the largest Danish cities in the eastern part, as well as rural areas in the western part of the region. The stratification furthermore ensures a minimum of time span between randomisation and invitation as local screening units individually can report back to the central randomisation office about their time schedule.

Person-data are imported to Epidata and Epi-info 6, where randomisation occurs by giving each subject a random number from 1-100, persons with a number above 51 are allocated to the intervention arm i.e. half of subjects are invited to participate in screening and half of subjects act as controls (as no permanent screening programme exists in Denmark).

### Outcome

The primary outcome variable is all-cause mortality. The secondary outcome variables are cardiovascular mortality, AAA-related mortality, hospital services related to cardiovascular conditions and costs for such services, quality of life, and aneurismal progression. Major follow-up is planned after 3, 5 and 10 years. All-cause mortality, health care-related costs, cardiovascular and AAA-related mortality and cardiovascular hospital service are compared for the two groups.

### Analysis of efficacy

Statistical analysis will be performed on an intention to treat basis. Risk ratios for all-cause mortality, cardiovascular mortality, AAA-related mortality and first cardiovascular event in the two groups will be estimated by Cox-regression. In addition, the numbers of hospital services related to cardiovascular conditions will be compared between the two groups by student's t-test. Quality of life between controls, non-attenders, attenders without positive finding, and attenderswith AAA or PAD will be compared at baseline univariately and multivariately adjusted for age, civil status and co-morbidity, and prospective data from baseline, after 3, 5 and 10 years will be compared by MANOVA. Aneurysmal progression (growth rate) will be transformed by the natural logarithm and evaluated in subgroups by univariate and multivariate linear regression analysis. SPSS 15.0, STATA 12.0 and PEPI will be used for the analysis.

### Analysis of cost-effectiveness

The program cost per invited will be estimated using a micro-costing approach for the intervention (invitations, screenings, follow up scans). Relevant resource utilization at hospitals (surgical procedures, admissions, outpatient visits etc.) will be extracted from the National Patient Registry and valued using tariffs of the Diagnosis-Related Grouping (DRG) case-mix system. Participants' time and transportation costs will be estimated from a prospective within-sample study of about 1,000 participants using a questionnaire.

Health-related quality-adjusted life will be estimated for each individual assuming that baseline quality of life follows that of an age- and sex-matched population norm [17]. For persons detected with and/or treated for cardiovascular disease estimates of decrements in quality of life will be adapted from the literature and applied.

The net present values of costs, life years gained and QALYs gained will be compared between randomization groups [18]. The incremental ratios of costs per life-year gained and costs per quality-adjusted life year (QALY) gained will be estimated using nonparametric bootstrapping; for their interpretation the net benefit framework will be used to present cost-effectiveness acceptability curves [19,20].

### Power and expected benefit

The needed sample size estimation was done by the following assumptions:

The prevalence of AAA and PAD is 4% and 8% respectively. The five year mortality risk in case of AAA or PAD is 28% oppose to 15% in the control group. Statin-lowering or antiplatelet medication can be initiated in one-third of the diagnosed men with PAD or AAA and causes a relative all cause mortality risk reduction of 20%. AAA-related death within five years is 1% but screening reduces this with 50%. The expected screening attendance is 75%

If so, the needed sample size is 2 × 23,604 (5% significance level and 90% power) and approx. 250 lives will be saved. If the ten year mortality in the control group is 36% [3], and if the relative risk is maintained, the needed sample size is 2 × 7,500, corresponding to 2.5% absolute mortality risk reduction and 590 lives saved corresponding to 40 men needed to screen to prevent one death.

These calculations must be considered to be conservative and the potential benefit by diagnosing new hypertensives is not included.

### Ethical considerations

This trial is approved by the regional scientific ethics committee and by the national data protection authorities. In accordance with the Clinical Trial Registration Statement from the International Committee of Medical Journal Editors, the trial is registered at clinical trials with the registration number NCT00662480.

## Discussion

The study population of men aged 65-74 years has been selected due to its relatively high cardiovascular risk, as most studies have reported increased risk with increasing age and lower prevalences of PAD and AAA in women. Nonetheless recent studies [21,22] indicates that the incidence of PAD in women is also considerable especially among elder women. Data on the prevalence and prognosis in woman are however too sparse at the moment to justify a parallel randomised trial but indeed it may be relevant to include women in a later stage, especially if the existing trial proves to be cost effective.

## Conclusions

The VIVA screening trial is a prospective randomized clinical trial examining the efficacy and cost effectiveness of a screening programme for early detection of AAA and PAD across 50,000 men aged 65-74 years. The primary outcome is all-cause mortality and secondary outcomes are AAA-related mortality, costs of hospital services related to cardiovascular conditions, quality of life, aneurismal progression and cost-effectiveness. Upon evaluation of outcome parameters after 3, 5, 10 and 15 years, this study will inform policy-makers about the attractiveness of introducing a national, permanent screening programme for cardiovascular disease

## Competing interests

The authors declare that they have no competing interests.

## Authors' contributions

All of the authors contributed equally and approved to this study protocol.
